# Prolonged Exposure to Platelet Activating Factor Transforms Breast Epithelial Cells

**DOI:** 10.3389/fgene.2021.634938

**Published:** 2021-03-25

**Authors:** Vaishali Chakravarty, Libi Anandi, K. A. Ashiq, K. Abhijith, Rintu Umesh, Mayurika Lahiri

**Affiliations:** Department of Biology, Indian Institute of Science Education and Research, Pune, India

**Keywords:** platelet activating factor, transformation, breast cancer, epithelial-mesenchymal transition, polarity

## Abstract

Lipid species are known to have various biological functions owing to their structural differences, and each of them possesses a specific role to play depending upon their location and distribution in the cell. Some of these lipids interact with proteins on the cell membrane and acts as second messengers. The level of lipid mediators is generally maintained in the cell by feedback mechanisms; however, their improper degradation or enhanced production leads to their accumulation in the tumor microenvironment and disturbs the homeostasis of the cell. Platelet activating factor (PAF) is a known phospholipid mediator secreted upon immunological challenges by platelets, neutrophils, basophils, and macrophages. PAF, as a potent inflammatory molecule, is well studied, and its role in various cancers and cardiovascular diseases has also been investigated. Interestingly, increased levels of PAF have been found in the blood plasma of smokers, and breast cancer cells have shown the accumulation of PAF in presence of cigarette smoke extract. This accumulation was found to increase tumor cell motility that in turn could promote metastasis. Beyond this, however, the effect of PAF on tumorigenesis has not yet been well explored. Here, we show that the continuous exposure of 3D breast acinar cultures to PAF resulted in the activation of various oncogenic signaling pathways leading to transformation. We also found that the presence of PAF in the micro-environment increased the expression of PAF receptor (PAF-R), which corroborated with the higher expression of PAF-R detected in some epithelial cancers, as per literature. Thus, this study impresses on the fact that the presence of PAF alters the cellular microenvironment and eventually triggers irreversible effects that can cumulatively lead to transformation.

## Introduction

Immune system holds a key position in maintaining cellular homeostasis, and an imbalance can lead to various abnormities including cancer. Hence, it is one of the primary targets while designing therapy for malignancies. Tumor infiltrating cells, such as leukocytes and macrophages, are common entities in cancers, and their interaction with the tumor micro-environment components is widely studied (Hanahan and Weinberg, 2011). Such entities are known to promote tumor progression and invasion, and thus hold a clinical relevance in various cancers (Man et al., 2013). The interaction of the immune cells and the cancer cells could be through direct cell-cell contact or may also be mediated by secretory molecules. One such molecule is platelet activating factor (PAF). PAF is an autacoid phospholipid mediator, which is secreted in response to an agonist by various immune cells and elicits various immunoregulatory reactions such as platelet aggregation, allergy, anaphylaxis, and many more (Foa et al., 1985; Hanahan and Weinberg, 2011). Overall, it is known to induce various physiological and pathological processes that eventually affect the respiratory, vascular, and even reproductive system (Papakonstantinou et al., 2017). PAF production is tightly regulated by biosynthetic and degradative mechanisms since uncontrolled levels can cause various pathologies. PAF acetyl hydrolase (PAF-AH) controls the level of PAF in the system by degradative mechanisms (Chen et al., 2007).

In the early studies, after the role of PAF was established in various pleiotropic activities, its effects were observed in various diseases as well (Lordan et al., 2019). PAF-AH activity was seen to be inhibited when human plasma was treated with cigarette smoke extract (CSE). This was also strengthened by the results that showed increased plasma concentration of PAF in smokers, indicating the role of PAF in the development of cardiovascular diseases in smokers (Miyaura et al., 1992). Kispert et al. (2015) has shown that CSE inhibits PAF-AH activity, thus leading to the accumulation of PAF in the endothelial cells. In a recent study with a bladder cancer cell line, the same group has reported similar results, wherein grade III HT-1376 cells showed higher PAF accumulation followed by greater adherence in presence of CSE, exhibiting highly aggressive manifestations (Kispert et al., 2019). Investigations by Bussolati et al. showed the presence of high amounts of PAF in MCF-7, MDA-MB 231, and T47D cells. This synthesized PAF enhanced cell motility as well as increased proliferation in MDA-MB 231 cells (Bussolati et al., 2000). An early report from Bennett et al. demonstrates that PAF induces phenotypic transformation in rat embryonic cells. They have shown increased cell density, growth in low serum condition, and anchorage-independent growth, which are the indicators of transformation, in the presence of dose-dependent increase in PAF concentration (Bennett et al., 1993).

Although PAF is a lipid mediator, it is not known to freely diffuse through the cell membrane and studies clearly state that PAF activates a G-protein coupled receptor, PAF-R and mediates its activities through this axis. PAF-R is ubiquitously expressed across tissues, and its role in various cancers has been established (Ishii and Shimizu, 2000; Jancar and Chammas, 2014). The PAF-PAFR axis activation suggests a feedback control loop that maintains the PAFR levels in pathological conditions (Chen et al., 2015a; Lv et al., 2017). Two groups in 2015 have shown the role of PAFR in non small cell lung carcinoma (NSCLC) and esophageal squamous cell carcinoma (ESCC). In NSCLC, the activation of PAF-PAF-R axis induces epithelial-mesenchymal transition (EMT), leading to invasion and metastasis of NSCLC cells (Chen et al., 2015a). Role of PAF-R in ESCC malignancy has been stated *via* the activation of oncogenic signaling through FAK/PI3K/AKT/NF-kB axis (Chen et al., 2015b). Previous report from the lab has shown that breast cancer cells in the presence of PAF shows higher motility and when MCF10A, a non-tumorigenic breast epithelial cell line, was grown on an ECM, in presence of PAF, disrupts the luminal phenotype (Anandi et al., 2016). Results from the lab as well as mounting evidence from literature imposed heavily on the necessity to investigate the role of PAF in the transformation of three-dimensional (3D) cultures of MCF10A cells. In the current study, we have focused on elucidating the transformation phenotypes acquired by MCF10A breast acinar cultures in the presence of PAF and the activation of PI3-K/AKT signaling pathway to strengthen the oncogenic potential of PAF.

## Materials and Methods

### Cell Lines and Culture Conditions

MCF10A cell line was a generous gift from Prof. Raymond C. Stevens (The Scripps Research Institute, California, United States) and 2D monolayer cultures and 3D cultures were grown according to the standard protocols (Debnath et al., 2003). 3D cultures were maintained for 20 days by supplementing fresh media every 4 days. Methylcarbamyl PAF C-16, procured as a 10 mg/ml solution in ethanol (Cayman chemicals, 60908), was diluted in sterile PBS to make a working stock of 100 μM. Required volume of the working stock was directly added to the desired volume of media, to achieve a final concentration of 200 nM. PAF treatment was given 3 h post seeding and along with every media change.

### Immunoflourescence

Acini from the 3D cultures were extracted using PBS-EDTA treatment for apical proteins and other proteins using normal method of extraction. Immunostaining was done using a previously described protocol (Anandi et al., 2017). Samples were imaged using 40X oil immersion objective of SP8 confocal microscope (Leica, Germany).

Details of chemical, antibodies, and statistical analysis used and methods for 3D “on top” cultures, immunoblot analysis, RNA Extraction, cDNA preparation, semi-quantitative PCR, soft agar assay, DQ collagen invasion assay, and gelatin zymography can be found in Supplementary Material.

## Results

### PAF Treatment on 3D Cultures of MCF10A Leads to Increased Proliferation

**Figure 1:**
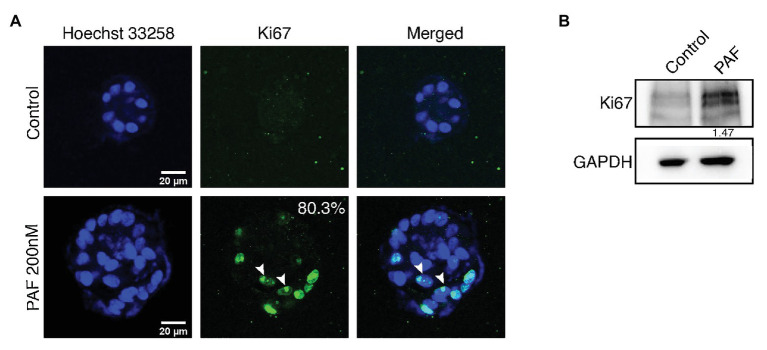
PAF treatment on 3D cultures of MCF10A leads to increased proliferation. MCF10A cells grown as 3D cultures for 20 days treated with and without PAF (200 nM) were immunostained with the proliferation marker, Ki67. **(A)** Representative image showing hyperproliferation in PAF-treated acini. The data are from *N* = 5 set of experiments. **(B)** Protein lysates from 20-day cultures were immunoblotted for Ki67. The values represent the relative expression of Ki67 normalized to GAPDH.

3D breast acinar cultures, grown on laminin rich matrix, attain a growth arrested state with a monolayer of cells around a hollow lumen, after 12 days of culturing. Cells in such stable structures are usually arrested in the G1 Phase (Fournier et al., 2006). Based on our previous data (Anandi et al., 2016), to ascertain if PAF treatment resulted in the presence of hyperproliferating and actively dividing cells, that resulted in large acini, we immunostained the PAF-treated 20-day cultures with Ki67, a proliferation marker. Acini with more than five cells positive for Ki67 are considered to be hyperproliferating (Wu and Gallo, 2013). Eighty percent of the PAF treated acini were found to be hyperproliferating (Figure 1A). Further, immunoblotting revealed a 1.4-fold upregulation in Ki67 protein levels (Figure 1B). This shows that PAF leads to the formation of large multiple layered acini that have evaded the growth-arrested state and are hyperproliferating thus qualifying as a transformation phenotype.

### Apico-Basal Polarity and Cell-Cell Junctions Gets Disrupted in the Presence of PAF Treatment

**Figure 2:**
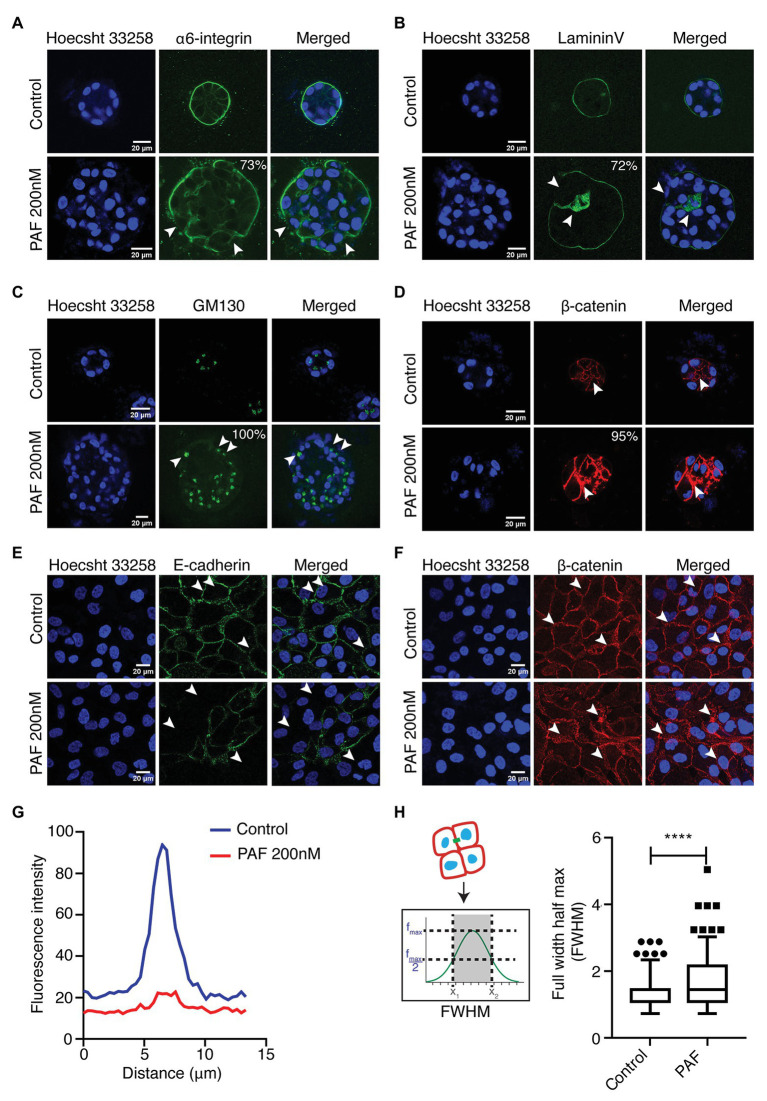
PAF treatment alters acinar polarity and leads to the disruption of cell-cell junctions. PAF-treated 20 days 3D cultures of MCF10A were immunostained with polarity and cell-cell junction markers. Nucleus is stained with Hoecsht 33,258 (blue). **(A)** Immunostained images of α6-integrin (green) marks the basal polarity (*N* = 4). **(B)** Immunostained images of LamininV (green) marks the basement membrane of the acini (*N* = 4). **(C)** Immunostained images of GM130 (green) marks apical polarity (*N* = 3). **(D)** Immunostained images of β-catenin (red) marks the cell-cell junctions. **(E)** Dissociated cells of control and PAF-treated acini immunostained with E-cadherin (green), a cell-cell junction marker and the image is a representative of the phenotype (*N* = 3). **(F)** Dissociated cells of control and PAF treated acini immunostained with β-catenin (red), a cell-cell junction marker and the image is a representative of the diffused phenotype (*N* = 3). **(G)** Median fluorescent intensity of E-cadherin line profile showing loss phenotype. **(H)** FWHM profile of β-catenin line profile showing diffused phenotype. The phenotypes are quantified from *N* > 3 set independent experiments. Statistical analysis was performed for **(H)** using Mann Whitney U test; ^*****^p < 0.0001.

One of the important features of epithelial architecture is polarization. Epithelial cells attain apico-basal polarity when placed in a 3D environment unlike cells on a 2D plane, which only have front and rear polarities. Alongside, cell-cell junction is an important component in maintaining the epithelial tissue homeostasis. In this study, it is evident that PAF affects the orientation of epithelial polarity and leads to compromised cell-cell junctions. There are various proteins that are known to act as markers of polarity such as integrins, laminins, and also Golgi (Debnath et al., 2003; Debnath and Brugge, 2005). E-cadherin is an adherens junction protein, which is well-established in MCF10A unlike the tight junctions, which are poorly developed (Ivers et al., 2014). E-cadherin is a central component of the zona adherens junction complex, and the loss of E-cadherin is a key indicator of tumor aggressiveness (Kourtidis et al., 2017). The 3D cultures of MCF10A treated with PAF were extracted after 20 days and immunostained with apical and basal markers such as GM130, α6-integrin, and Laminin-V. α6-integrin, receptors for the ECM, marks the basal region of the acini and stains across the basolateral regions. In the presence of PAF, the staining pattern was altered dramatically by change in expression level as well as membrane distribution of the protein (Figure 2A). Seventy-three percent of acini treated with PAF exhibited marked loss of α6-integrin from the basal region and also enhanced cytoplasmic staining was observed, which we quantified as loss and mis-localized phenotype. Epithelial cells have a remarkable property of attachment to the ECM *via* the basement membrane (BM). This BM is disrupted in the cases of primary metastasis in breast carcinomas (Shaw et al., 2004). Laminins are a group of BM proteins and LamininV is one of the components of BM (Shaw et al., 2004). Acini grown in the presence of PAF showed gross disruption in LamininV staining, thus indicating loss of BM protein due to PAF treatment. Seventy-two percent of acini show disrupted as well as the loss of membrane localization of LamininV (Figure 2B). GM130, which is a cis-Golgi marker, was mislocalized to the basal and lateral regions in PAF-treated acini, whereas in the control acini Golgi localization remained apical. Upon quantifying, it was observed that 100% of Golgi was mislocalized in PAF-treated acini (Figure 2C). These results indicate a clear loss of apical as well as basal polarity in MCF10A 3D cultures grown in the presence of PAF. Immunostaining for β-catenin revealed a diffused localization of β-catenin at cell-cell junction as well as in intercellular granular pockets, which is also indicative of aberrant protein trafficking (Figure 2D). To further confirm the disruption of the cell-cell junctions, cells from day 20 spheroids were dissociated (referred to as dissociated cells hereafter) and immunostained for E-cadherin and β-catenin. Loss of E-cadherin by immunostaining was observed in the presence of PAF (Figure 2E) and analyzed using intensity plot profiling (Figure 2G). β-catenin also showed similar diffused patterns in the dissociated cells as was observed in the acinar cultures (Figure 2F). The diffused pattern was analyzed using a plot profiling of lines drawn across cell-cell junctions and then calculating Full Width Half Max (FWHM) as depicted in the schematic in Figure 2H. The FWHM values were significantly higher in PAF exposed cells as compared to untreated dissociated cells (Figure 2H). This clearly indicated that PAF treatment altered epithelial polarity and also led to a loss and/or disruption of cell-cell junctions.

### PAF Treatment Induces Partial EMT-Like Phenotype

EMT is a process of transition of epithelial cells into mesenchymal cells. This program has been distinctively viewed as a two-stage program with two discrete cell populations of epithelial and mesenchymal cells expressing the cell-type specific proteins (Pastushenko and Blanpain, 2019). However, recent studies indicate that EMT is a gradual process that takes place through stages that are known as intermediate states. This state circumscribes the co-expression of epithelial and mesenchymal markers. It has also been shown in similar studies that this hybrid/incomplete state of EMT is more culpable to poor survival and higher chances of resistance to therapy (Lambert et al., 2017; Pastushenko and Blanpain, 2019).

**Figure 3:**
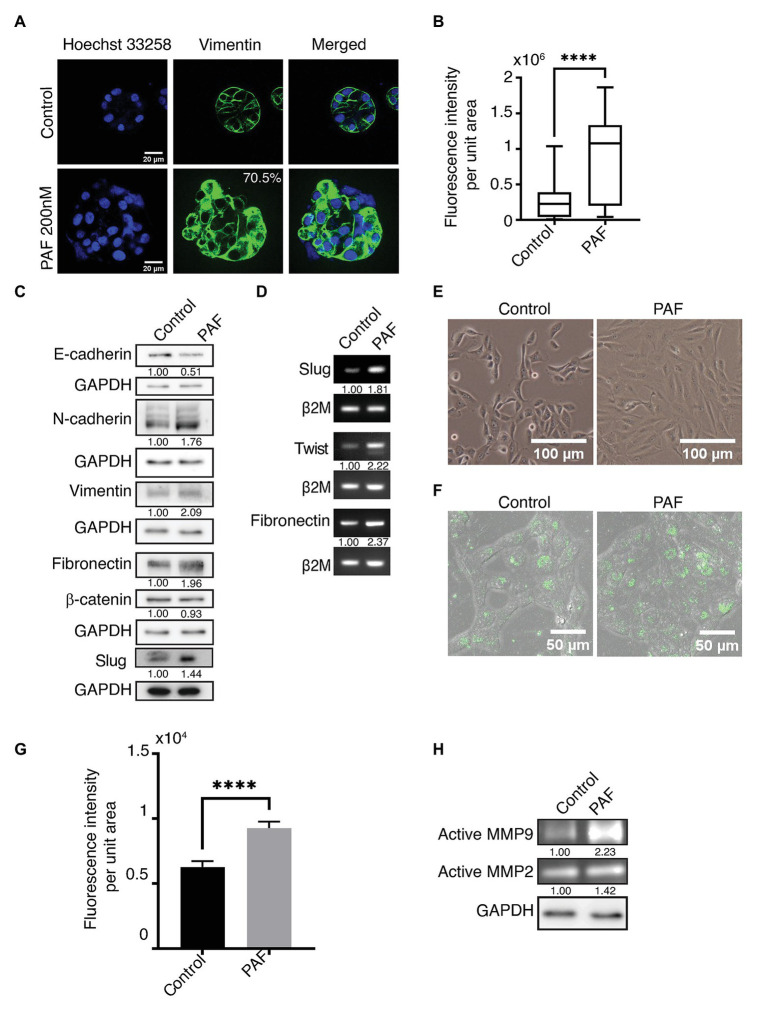
PAF treatment induces partial epithelial-mesenchymal transition (EMT)-like phenotype. MCF10A cells grown for 20 days on Matrigel™ treated with and without 200 nM PAF immunostained and immunoblotted with EMT markers. **(A)** PAF-treated acini showed upregulated vimentin expression (green). **(B)** Box plot depicts the acinar fluorescence (CTCF) of vimentin (*N* = 4). **(C)** 20-day 3D culture lysates of control and PAF immunoblotted for various EMT markers. The values represent fold change with respect to control and normalized to GAPDH (*N* = 3). **(D)** mRNA expression analysis done for various EMT markers using RNA lysates. The values represent fold change with respect to control and normalized to β2M (*N* = 3). **(E)** Phase contrast images of dissociated cells of PAF treated acini grown as 2D cultures showed a mesenchymal phenotype. **(F)** Dissociated cells of control and PAF, seeded on rat tail collagen type1 containing DQ™ collagen type1 and fluorescence intensity captured on SP8 confocal microscope (Leica, Germany). **(G)** Quantification of the DQ fluorescence per cell (*n* = 150). **(H)** Coomassie-stained gel showing gelatinase activity using conditioned media from 3D cultures. The values represent fold change with respect control and normalized to GAPDH. Statistical analysis was performed for **(A)** and **(F)** using Mann Whitney U test; ^****^p < 0.0001.

Vimentin, a known mesenchymal marker, showed an upregulated phenotype in the acini grown in the presence of PAF (Figures 3A,B). The protein expression of the different EMT markers was also investigated as shown in (Figure 3C). Vimentin and N-cadherin protein levels showed an upregulation in the presence of PAF, and densitometric analysis showed a 2-fold and 1.76-fold increase in the respective protein levels. Fibronectin, which is also a mesenchymal marker, is also upregulated in the presence of PAF. E-cadherin, an epithelial and a cell-cell junction marker showed a 0.5-fold reduction in protein level, which further corroborated the loss that was observed earlier (Figure 2E). β-catenin protein levels did not show a significant change probably because there has not been a major loss of the protein but more of cellular redistribution from the junctions to the cytoplasm as granules. Hence, a clear difference in protein level was not observed. Slug, a transcription factor, showed a 1.47-fold increase at protein level.

Transcript levels of the various EMT markers were also investigated in the 20-day PAF-treated 3D cultures. Fibronectin, slug, and twist transcript levels were measured using semi-quantitative PCR. Fibronectin transcript level was more than 2-fold higher in the RNA lysates collected post 20 days of PAF treatment. The transcript levels of slug and twist also showed a 2-fold upregulation in the presence of PAF (Figure 3D). The quantifications of the transcript level were performed using densitometric analysis using ImageJ software. The role of tumor micro-environment (TM) is well-established in various cancers including breast cancer (Yu and Elble, 2016). TM is composed of different inflammatory and immune cells, apart from other components, which have been known to induce EMT-like phenotype (Yu and Elble, 2016). As mentioned before, PAF is a phospholipid mediator that is widely known to play a role in the activation of various immune cells. Hence, the above results are indicative of the formation of a link between hybrid state of EMT, TM and PAF; all leading to transformation.

Figure 3E shows the phase contrast images of control and PAF-treated dissociated cells grown as monolayer cultures. Control cells appear like MCF10A cells, epithelial-like, and cuboidal, while PAF-treated cells appear spindle-like, large, and elongated. This appearance is retained when cells are both sparsely or densely seeded. This morphology strengthens the fact that PAF has the ability to transform MCF10A breast epithelial cells.

Since the data indicate induction of EMT, this was further supported by performing DQ collagen™ assay. PAF dissociated cells showed increased fluorescence when observed under SP8 confocal microscope (Leica, Germany; Figure 3F), which was quantified (Figure 3G). To strengthen the hypothesis that PAF induces invasion, gelatin zymography was performed using the conditioned media (CM) from the 3D breast acinar cultures. Increased activation of MMP-2 (1.42-fold) and MMP-9 (2.23-fold) were observed in the conditioned media collected from the PAF-treated samples (Figure 3H), thus indicating the ability of PAF stimulation to induce invasion, which is a known marker for transformation. Taken together, our data clearly indicate the induction of partial EMT and invasion in PAF-treated cells thereby leading to a transformation phenotype.

### Effect of PAF on Anchorage Independent Growth of MCF10A Cells and Activation of the PI3K/AKT Pathway

**Figure 4:**
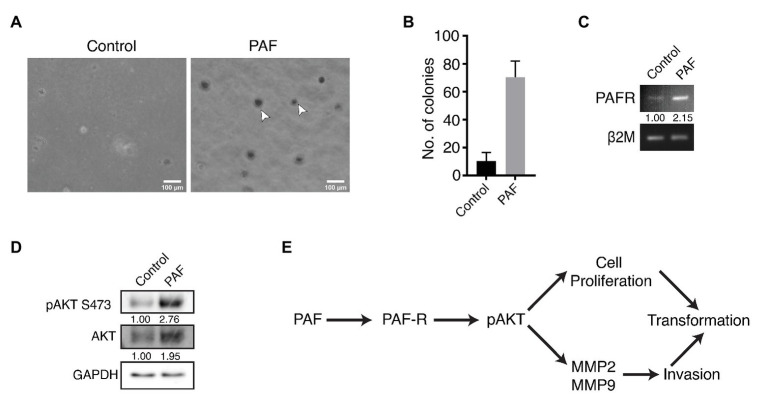
Effect of PAF on anchorage independent growth of MCF10A cells and activation of the PI3K/AKT pathway. Dissociated cells were grown on soft agar and stained with MTT to visualize colonies formed and the pathway for PAF induced transformation. **(A)** Phase contrast images showing MTT stained colonies grown on soft agar. **(B)** Quantification of the number of colonies formed on soft agar (*N* = 3). **(C)** Transcript level of PAF-R. The values represent fold change with respect to control and normalized to β2M (*N* = 3). **(D)** Western blot indicating activation of AKT through PAF-R. The values represent fold change with respect to control and normalized to GAPDH (*N* = 3). **(E)** Pathway showing the activation of AKT downstream of PAF-R activation.

Anchorage independent growth is a key characteristic feature that cells attain when they have undergone transformation, and this ability is considered as a fundamental property of cancer cells. This capacity to grow on a semisolid substratum serves as a proxy for *in vivo* tumorigenicity (Horibata et al., 2015). In our study, soft agar assay was performed to investigate whether prolonged PAF-treatment could transform the cells, thus allowing them to grow in an anchorage-independent manner. Dissociated cells of both control and PAF-treated acini were grown on soft agar for 27 days and then stained with MTT, a tetrazolium dye, to visualize the colonies and to distinguish the dead cells from live ones. Figures 4A,B clearly show PAF dissociated cells to have gained the capability to form colonies in an anchorage-independent manner, which supports the hypothesis that PAF induces *in vitro* tumorigenesis.

Our data indicate PAF stimulation of non-tumorigenic breast epithelial cells to undergo transformation; however, the mechanism of this transformation process is not known. In the current study, PAF-R showed a 2-fold upregulation in transcript levels of the PAF-stimulated lysates (Figure 4C). Further, PAF-treated 3D culture lysates were immunoblotted and probed for pAKT and total AKT. pAKT showed close to 3-fold increase in activation with concomitant increase in AKT protein expression upon prolonged treatment with PAF (Figure 4D).

In support of the existing literature, our data also show MMP-2 and MMP-9 activation downstream of pAKT activation. Increased cellular proliferation observed in the presence of PAF is suggestive of the contribution of the AKT pathway in the PAF-mediated transformation of MCF10A cells grown as 3D cultures. Hence, we predict the following pathway (Figure 4E) using existing data and current literature; however, further research is required to delineate the mechanism of transformation through PAF. Since our data are preliminary, further studies are called for using small molecule inhibitors to thoroughly dissect the signaling mechanism. Overall, PAF leads to phenotypic transformation of breast epithelial cells when grown on ECM for 20 days and the activation of the oncogenic molecular players warrants deeper investigations for the mechanism.

## Discussion

PAF is long known as a potent inflammatory bioactive molecule that is unequivocally proven to give rise to aberrant physiological effects (Lordan et al., 2019). Its role in various types of cancer is studied; however, the mechanism that leads to cancer is still understudied (Tsoupras et al., 2009). The current study will be instrumental in defining the mode of action of PAF in transforming non-tumorigenic breast epithelial cells. Health hazards of high levels of PAF in the system were studied earlier by Kispert et al., and although it is known to be naturally present in the living system, improper degradation leads to its accumulation and hence leads to cellular and molecular anomalies (Chen et al., 2007; Kispert et al., 2015, 2019). Bussolati et al. (2000) have shown that the stimulated secretion of PAF promotes migration, proliferation, and neo-angiogenesis in breast cancer cells, clearly indicating its role in carcinogenesis (Bussolati et al., 2000). Cell microenvironment perturbations by exogenous or endogenous sources lead to increased cellular proliferation that results in a loss of hollow lumen followed by the formation of multiple layers of cells in the lumen. Such a phenotype is predictive of transformation and can be a representative model for early stage mammary cancer *in vitro* (Vidi et al., 2013). Formation of a polarized, growth-arrested acinar structure with glandular epithelial cells surrounding a hollow lumen is archetypal of perfectly synchronized cellular and molecular events culminating into a normal breast acinus. When this tissue architecture is disturbed, cell survival, and differentiation processes get largely affected and eventually pave the way to tumor formation (Vidi et al., 2013). Transformation is the initial step to this process, wherein the normal cell signaling goes haywire, and there is an aberrant expression of genes leading to malignant phenotypes, and this phenomenon is nearly recapitulated using the 3D breast acinar model system. Formation of disorganized acinar structures is characteristic of transformation and various phenotypic changes are categorized under lack of growth arrest, loss of cell polarity, disruption of BM and cell-cell junctions, EMT, and ability to grow in anchorage-independent conditions (Debnath et al., 2003). Our study encompasses all the above phenotypes in the zest to investigate the role of PAF in transformation of breast epithelial cells using the 3D breast acinar model.

Investigations revealed that continuous PAF treatment for 20 days leads to the disruption of polarity manifested by loss of apico-basal polarity. Generally, for apical polarity, *in vitro* studies as well as pathologists utilize Golgi marker as a strong identifying marker to understand polarity alteration leading to transformation or cancer (Debnath et al., 2003). Golgi is the hub for signal transduction for various pathways and alteration in its localization affects the polarized secretion of proteins from the ER to the plasma membrane and other compartments that in turn affects the signal transduction pathways (Millarte and Farhan, 2012). Hence, although the global secretory pattern does not change, but the directed trafficking of cargo is impaired. Alongside, breakage of BM is the key to loss of basal polarity. This phenomenon is significant because it provides a hallway to invading cells into the surrounding ECM and further into tissue stroma. The entirety of the BM is necessary to limit tumor growth and invasion and for this reason many solid tumors show discontinuous or a loss of BM (Ioachim et al., 2002). These investigations and the supporting literature revealed a structural loss of tissue architecture and loss of epithelial polarity in the presence of PAF, indicating that such phenotypes are important components toward the maintenance of polarity and disruption of these leads to transformation and gives direct evidence in cancer development and progression (Lee and Vasioukhin, 2008).

E-cadherin, a cell-cell junction protein is known to maintain epithelial polarity by being a center for the intercommunicating signals between catenins and the cytoskeletal network and disruptions in this contact lead to various morphogenetic and developmental defects apart from loss of epithelial polarity (Olson et al., 2019). E-cadherin complexes with cytoplasmic β-catenin at the junctions and aberrations in this interaction leads to an increased flux of cytoplasmic β-catenin resulting in transformation and such an observation is commonly noted in solid tumors and epithelial cancers (Kourtidis et al., 2017). In our study, the loss of E-cadherin and diffused β-catenin at the junctions was observed in cells treated with PAF. Dissociation of the E-cadherin and β-catenin proteins from the complex leads to aberrant expression at the cell membrane and attributes to malignant progression, as well as has importance as a prognostic marker (Kaur et al., 2013). β-catenin expression leads to the transcription of various EMT inducing genes, thus identifying the various proteins that are under its transcriptional activity that lead to EMT is important (Solanas et al., 2008). EMT is a biological process that is employed during developmental stages; however, carcinoma cells hijack this program and herein the cells tend to lose their epithelial-like characteristics, both morphologically and functionally, and become mesenchymal or express such characteristics. Induction of EMT leads to the activation of various oncogenic programs including enhanced migration, invasion, metastasis, and the evasion of apoptotic stimuli (Kalluri and Weinberg, 2009; Scheel and Weinberg, 2011). Such complex programs are orchestrated by various EMT regulating transcription factors such as Snail, Slug, Twist, Zeb1, to name a few, to culminate into EMT. Apart from transcription factors, loss of cell-cell contacts and aberrant polarity also triggers EMT initiation. As cells fall loose in the cell matrix, they degrade it and attain mobility and this process is an attribute to EMT, which leads to the expression of mesenchymal proteins (Lamouille et al., 2014; Puisieux et al., 2014). Considering SNAIL1 and TWIST, the master regulators of EMT, they are classically known to repress E-cadherin and this opens the gate for tumor cells to migrate, either by mesenchymal movement or by amoeboid movement (Padmanaban et al., 2019). Concurrently, as E-cadherin levels decrease, N-cadherin levels increase and literature suggests that N-cadherin expression is TWIST-dependent (Alexander et al., 2006). Apart from the typical EMT markers, there are other molecules as well that play important roles in pathological EMT pathways. Fibronectin is an extracellular matrix protein, which gets enriched when EMT is induced, as well as various MMP’s are expressed, which aid in the degradation of the matrix and enable tumor cells to invade deeper into tissues. Studies show that MMP-2 and MMP-9 deficient mice show impaired tumor cell growth and poor metastasis (Itoh et al., 1998). So, the conjoint efforts of BM disruption, mesenchymal expression, and MMP activation hold key position in inducing EMT, and the study suggests the same. However, inherent heterogeneity that arises in carcinoma cells, the tissue-wise difference in signal that cells receive to drive the EMT program, the signaling pathways that get activated in response to the induction of EMT, and the original state of the normal differentiated cells are few of the factors that deter making a perfect molecular trait chart that describes EMT (Lambert et al., 2017).

Traditionally, anchorage independent growth is a key feature of a transformed cell, and this phenomenon is well studied in the limited scope of *in vitro* culture system through colony formation in low attachment conditions. In our study, we have shown that acinar cultures grown in the presence of PAF attain anchorage independent growth, when grown on soft agar. As a first attempt these investigations gave enough evidence to show that PAF leads to transformation and the molecular players involved in this process is probably pAKT (S473; Chen et al., 2015b). Further studies are necessary to delineate the pathways that are involved in transformation through PAF.

Until now several investigations have shown that PAF plays a leading role in inflammation, platelet aggregation, leukocyte migration, and many such inflammatory mechanisms (Chignard et al., 1979; Bussolati et al., 2000). Cigarette smoke exposure is shown to promote bladder cancer and motility leading to metastasis in breast cancer cells *via* PAF (Kispert et al., 2015, 2019). In our study, we show that continuous exposure of PAF to 3D breast acinar cultures for 20 days leads to the formation of aberrant acinar morphology as well as the functional disruption of acinar morphogenesis, which might be due to activation of the PI3K/AKT pathway.

## Data Availability Statement

The original contributions presented in the study are included in the article/Supplementary Material, further inquiries can be directed to the corresponding author.

## Author Contributions

LA, VC, and ML: study conceptualization, supervision, study design, and wrote the paper. LA, VC, KAA, KA, and RU: data collection. All authors contributed to the article and approved the submitted version.

### Conflict of Interest

The authors declare that the research was conducted in the absence of any commercial or financial relationships that could be construed as a potential conflict of interest.
