# Supplementary material for: Prolonged Exposure to Platelet Activating Factor Transforms Breast Epithelial Cells
Source: Front Genet. 2021 Mar 25;12:634938. doi: 10.3389/fgene.2021.634938 (PMC8027472; doi:10.3389/fgene.2021.634938)
Supplement: Supplementary file 1 [file Data_Sheet_1.pdf]

Supplementary Material

Material and Methods

Chemicals and antibodies:

Carbamyl PAF (Platelet Activating Factor) was procured from Cayman chemicals. Paraformaldehyde (16% w/v aqueous solution) used for immunostaining was bought from Alfa Aeser. DQ™ Collagen type1 was purchased from Invitrogen (D12060) while Collagen 1 Rat protein, tail was from Thermo Fisher Scientific (A1048301). Monoclonal antibodies used for immunofluorescence for laminin-5 (MAB19562) and α6-integrin (MAB1378) were bought from Millipore, GAPDH (G9545) antibody was bought from Sigma-Aldrich. Monoclonal antibodies for immunofluorescence for Ki67 (ab16667), vimentin (ab92547), β-catenin (ab32572), GM130 (EP8924) and E-cadherin (ab1416) were obtained from Abcam. For western blotting, monoclonal antibodies for Ki67 (ab16667), vimentin (ab8069) and N-cadherin (ab98952) was obtained from abcam. For western studies, fibronectin monoclonal antibody (610077), E-cadherin (610182), β-catenin (6101-53) were obtained from BD Biosciences. Polyclonal antibody for pAKT (S437) (9271S) was purchased from Bioresources. Slug (C19G7) (9585) monoclonal antibody was procured from Cell Signalling Technology. Peroxidase-conjugated AffiniPure goat anti-mouse and anti-rabbit as well as AffiniPure $F(ab')_2$ fragment goat anti-mouse IgG, $F(ab')_2$ fragment specific were obtained from Jackson Immuno Research. Hoechst 33258, Phalloidin 568 and 633, Alexa Fluor 488 and 568 were bought from Invitrogen.

3D "on top" cultures:

The 3D "on top" culture was set up in 8 well, 24 well and 12 well dishes using standard protocols (14,15,16). Cultures were maintained for 20 days at 37 °C and assay medium containing 2% Matrigel and 5 ng/mL EGF was supplemented every 4 days.
To dissociate cells from spheroids, cultures grown on 12 well dishes were treated with 187 µl of Dispase™ and incubated at 37 °C for 30 minutes. The solution was collected in tubes and centrifuged at 900 rpm for 10 mins, supernatant discarded and further resuspended in growth medium. Centrifugation was repeated and the pellet was then resuspended in growth medium and replated on a 12 well dish. After 48 hours, depending on confluency (approximately 70%) cells were trypsinized and replated and finally the culture was expanded and cells were frozen down at every passage and later used for the experiments.

Immunoblot analysis:

MCF10A cells were grown on Matrigel® for 20 days in presence of PAF and the spheroids were extracted using sample buffer containing 0.06 mM Tris (pH 6.8), 6% glycerol, 2% sodium dodecyl sulphate (SDS), 0.1 M dithiothreitol (DTT) and 0.006% bromophenol blue and lysates were stored at - 40°C. These lysates were resolved on SDS-PAGE (sodium dodecyl sulphate polyacrylamide gel electrophoresis) and transferred to PVDF (P-polyvinylidene difluoride) membrane (Millipore). Blocking for non-phospho antibodies was performed in 5% (w/v) skimmed milk (SACO Foods, USA) and for phospho-specific

antibodies 4% (w/v) Block Ace (AbD Serotec) prepared in 1X Tris buffered saline containing 0.1% Tween 20 (1X TBS-T) for 1 hour at RT. Blots were incubated in primary antibody for 3 hours at RT (or for 16 hours at 4 °C) followed by washes with TBS-T, blots were then incubated with peroxidase-conjugated secondary antibody solution in the ratio 1:10,000 prepared in 5% (w/v) skimmed milk in 1X TBS-T for 1 hour at RT following which blots were developed using Immobilon Western Detection Reagent kit (Millipore) and visualised using ImageQuant LAS 4000 (GE Healthcare). Densitometric analysis using Image J software was performed in order to calculate the relative expression of proteins in the western blots.

RNA Extraction, cDNA preparation and semi-quantitative PCR:

For extraction of RNA, MCF10A cells seeded on Matrigel® bed made in 12 well plates at a density of 1.25 X 10^5 per well were harvested on day20 by scrapping cells following addition of TRIzol (Ambion). RNA extraction was done using standard protocol. 1µg of RNA was used for preparation of cDNA using 1 µl oligo dT (50 mM; Invitrogen) in RNAse and DNAse free water, dNTP (2.5 mM) and M-MLV-reverse transcriptase (Invitrogen). 1µl of the resultant reverse transcribed mix was PCR amplified with Taq DNA polymerase using 0.2µM of respective primers, and 0.125mM dNTP mix. Amplification was done using the following PCR cycle: 95 °C for 60 sec, 55 °C for 45 sec, 72 °C for 60 sec and final extension for 5 min; 40 cycles. Densitometric analysis of the gel images were performed using Image J software to determine the relative expression of the transcripts. Primer sequences used are as listed in Table 1:

Gene of interest	Forward	Reverse
TWIST	CGGAGACCTAGATGTCATTG	ACGCCCTGTTTGTTTGAAT
SLUG	TGTTGCAGTGAGGGCAAGAA	GACCCTGGTTGCTTCAAGGA
PAFR	TACTGCTCTGTGGCCTTCCT	CTGCCCTTCTCGTAATGCTC
FN	CCCACCGTCTCAACATGCTTAG	CTCGGCTTCCTCCATAACAAGTAC
β_2M(qRT)	TGTCTTTCAGCAAGGACTGGT	CTGCTTACATGTCTCGATCCCA

Soft Agar assay:

1.25 x 10^5 MCF10A cells were mixed with 0.3% (w/v) agar in DMEM and seeded onto a tissue culture dish coated with 0.6% (w/v) agar in DMEM. This culture was incubated at 37°C in 5% CO_2 incubator. Cells were supplemented with growth media 2-3 hrs post seeding and every 4th day (Anandi et al., 2017). The culture was maintained for 27 days and stained using MTT (5mg/ml). Images were acquired on Nikon Eclipse TS-100 under 4X objective and colonies were counted manually.

DQ collagen invasion assay

The dissociated cells of MCF10A acinar cultures treated with and without PAF were seeded in 8-well chambers coated with ECM composed of Matrigel and Collagen in 1:1 ratio. ECM mix and DQ was used as per previously described protocol (Anandi et al., 2017). After the incubation period, cells were imaged at 40X magnification on SP8 confocal microscope (Leica, Germany). Fluorescence intensity from DQ cleavage was measured using Image J. Corrected total cellular fluorescence (CTCF) was calculated using the equation:

CTCF=Integrated fluorescence intensity of cell-(Area of cell*mean fluorescence intensity of background).

Gelatin Zymography:

After 20 days of culturing, the assay media from untreated MCF10A and 200 nM PAF-treated MCF10A cells were collected. The media was mixed with Gelatin Zymography (GZ) buffer and run on a 0.1% gelatin SDS-PAGE. The gel was developed using standard protocol (Hu and Beeton, 2010). The gel images were captured using ImageQuant LAS4000 gel documentation system (GE Healthcare) and densitometric analysis using Image J software was done on the gel images to ascertain relative increase in the secretion of MMPs.

Immunoflourescence Analysis:

Fluorescence intensity for acinar structures was quantified using Image J software and analyzed by calculating the corrected total cellular fluorescence (CTCF) as described previously. Dissociated MCF10A cultures stained for β-catenin and E-cadherin was analyzed using plot profile function of Image J, for a line drawn perpendicular to the cell-cell junctions. The intensity profile for ~150 cell-cell junctions across 3 independent experiments was calculated and the mean was plotted to demonstrate the loss of E-cadherin at cell-cell junctions. For β-catenin, the full width half max for each plot profile was manually calculated (~100 cell-cell junctions) and represented as a box plot (Mikhaylova et al., 2015; Brandenburg et al., 2018).

Full Width Half Max = $|X_2-X_1|$; where X_2 , X_1 are the values of X at $f_{max}/2$. f_{max} is the function of X.

Statistical analysis:

Mann-Whitney U test, a non-parametric test, was used to analyse statistical significance of the difference in full width half max profile of β-catenin line profile to interpret the diffused phenotype. Mann-Whitney U test was also used to analyse the statistical significance of fluorescence intensity per unit area per acini for vimentin and fluorescence intensity per unit area per cell for DQ™ Collagen invasion assay. $p<0.05$ was considered statistically significant. **** indicates $p<0.0001$. Graph Pad Prism software (Graph Pad Software, La Jolla, CA, USA) was used to plot the graphs and analyse data.

References:

Anandi, L., Chakravarty, V., Ashiq, K.A., Bodakuntla, S., and Lahiri, M. (2017). DNA-dependent protein kinase plays a central role in transformation of breast epithelial cells following alkylation damage. *J Cell Sci* 130(21)**,** 3749-3763. doi: 10.1242/jcs.203034.

Brandenburg, S., Pawlowitz, J., Fakuade, F.E., Kownatzki-Danger, D., Kohl, T., Mitronova, G.Y., et al. (2018). Axial Tubule Junctions Activate Atrial Ca(2+) Release Across Species. *Front Physiol* 9**,** 1227. doi: 10.3389/fphys.2018.01227.

Hu, X., and Beeton, C. (2010). Detection of functional matrix metalloproteinases by zymography. *J Vis Exp* (45). doi: 10.3791/2445.

Mikhaylova, M., Cloin, B.M., Finan, K., van den Berg, R., Teeuw, J., Kijanka, M.M., et al. (2015). Resolving bundled microtubules using anti-tubulin nanobodies. *Nat Commun* 6**,** 7933. doi: 10.1038/ncomms8933.
